# Health literacy among university students in Greece: determinants and association with self-perceived health, health behaviours and health risks

**DOI:** 10.1186/2049-3258-72-15

**Published:** 2014-05-23

**Authors:** Athanassios Vozikis, Kyriakos Drivas, Kostantinos Milioris

**Affiliations:** 1 Economics Department, University of Piraeus, 80, Karaoli & Dimitriou str, 18534 Piraeus, Greece

**Keywords:** Health literacy, Health behaviour, Health risks, Greece, University student

## Abstract

**Background:**

Health literacy is widely considered as a key determinant of health and a priority in the public health policy agenda. Low health literacy has been associated with poorer health states, broader inequalities and higher health systems’ costs. In the present study we bring into focus the functional health literacy among university students in Greece, researching and assessing mainly their ability to apply basic knowledge in a health context.

**Methods:**

The study was carried out during the period 15–30 April 2013, among a random sample of 1,526 students of 14 Higher Tertiary Public universities and Technological Educational Institutes in Greece. The objective of the study was to assess the functional health literacy among university students in Greece, adopting the short four-item comprehension test of Bostock and Steptoe. Summary statistics, correlations and regressions were used to assess the determinants of health literacy and the association with self-perceived health, health behaviours and health risks.

**Results:**

Economic factors, such as family income, demographic factors, such as gender, and health behaviours and risks, namely consumption of alcohol, smoking and physical workout are associated with the level of health literacy and health status of the participant. While the results of the study are consistent with previous work in this area, several findings worth further research.

**Conclusions:**

Though, health promotion interventions in Greece include health literacy as one of the basic pillars of the public health policy agenda, it is clear, that health literacy needs to become a key policy issue in Greece, mainly focusing in young ages, where healthy (or unhealthy) behaviours are established affecting the health through the life span.

## Background

Empowered citizens/patients in the 21st century, often confront barriers, set by the educational and health systems, in accessing, understanding and assessing information in order to make well-informed decisions concerning their health [1]. Though health literacy has been seen in many perspectives and has been defined in various ways, the most comprehensive definition refers to the health literacy as "*the degree to which individuals have the capacity to obtain, process and understand basic health information and services needed to make basic health decisions*" [2,3]. Recently, a broader and more inclusive definition has been given by the Health Literacy Score (HLS)-Eu Consortium, stating that "*Health literacy is linked to literacy and entails people’s knowledge, motivation and competences to access, understand, appraise and apply health information in order to make judgements and take decisions in everyday life concerning health care, disease prevention and health promotion to maintain or improve quality of life during the life course*" [4].

Health literacy is widely considered as a key determinant of health and a priority in the public health policy agenda [5-9]. Low health literacy has been associated with poorer health states, broader inequalities and higher health systems’ costs [10-13].

Main determinants of health literacy have been found to be education, age, migration, employment status and income [4,11,14].

In the present study we study and assess the functional health literacy among university students in Greece, researching and analysing mainly their ability to apply basic knowledge in a health context [15].

Though, it might seems a target group of *de facto* high education level and good health status, it is also the age group (18–24 year old), on which all health systems should watch over, reforming their public health preventing policies, to establish healthy behaviours and eliminate health risks [16-21].

## Methods

The study was carried out by trained postgraduate students during the period 15–30 April 2013, among a random sample of 1,526 students, aged 18–24 years old, in 33 Departments of 14 Higher Tertiary Public universities and Technological Educational Institutes in Greece, located in six major cities of the country.

Although there is no widely accepted framework for measuring health literacy, most instruments focus on assessing an individual’s (usually functional) health literacy [22-24]. So in our study, to assess the students’ health literacy level we adopted the short four-item comprehension test of Bostock and Steptoe [25]. The same test has been used in various studies [26,27]. Students, after an introductory briefing about the study scope, were asked to read for five minutes a fictitious medicine’s (PHARMAKO) simplified information leaflet. Then they were handled a questionnaire and they were asked within 5 minutes to provide answers to four comprehensive questions referring to the information leaflet. They were allowed (but not encouraged) to have a glance on the leaflet, as this was not the case of memorizing the leaflet content. This rapid health literacy assessing method was developed according to a conceptual framework that defines literacy as an ability to fulfil goal directed tasks, in this case in a health context [28]. Each correct answer scored 1 point, resulting in a health literacy score of 0 to 4. To assure the reliability of the study, neither the interviewers, nor the students, were aware of the medicine’s name.

From the questionnaire we also obtained data on sex, age, marital and occupation status, city of residence, various health behaviours and the perceived health status (using the visual analog scale). No personal identification data were acquired or recorded (such as name, Student ID, Social Security ID etc.), so as to assure the anonymity of the participating students.

Finally, all participating students signed the informed consent statement and gave their permission to use the questionnaire content for the research purpose and only.

### Estimated specification

The estimated specification is the following one:

(1) $$Y_{i}=f\;\left({\text{Male}}_{i},\text{Income}{\left[<1,100\right]}_{i},\text{Income}{\left[1,100-2,200\right]}_{i},{\text{Alcohol}}_{i},{\text{Workout}}_{i}\right)$$

where the dependent variable *Y*_*i*_ is either the health literacy (HealthLiteracy) or the self-perceived health status (HealthStatus) of the respondent, *i*. The variable *HealthLiteracy* is a discrete variable, which ranges from 0 (minimum health literacy grade) to 4 (maximum health literacy grade), a respondent can achieve. The *HealthStatus* ranges from 0 to 100*. Male* takes the value of 1 if the student is male and 0 otherwise. *Income[<1,100]* takes the value of 1 if the student’s family income is less than 1,100 Euros and 0 otherwise. *Income[1,100-2,200]* takes the value of 1 if the student’s family income is between 1,100 Euros and 2,200 Euros and 0 otherwise. There is also the variable *Income[>2,200]* which takes the value of 1 if the student’s family income is more than 2,200 Euros; this latter variable is not included in the specification to avoid the dummy variable trap. *Smoking* takes the value of 1 if the student is a frequent smoker and 0 otherwise. *Alcohol* takes the value of 1 if the student consumes alcohol daily or almost daily and 0 otherwise. *Workout* takes the value of 1 if the student takes the value of 1 if the student works out more than once a week. Note that in the case where we explore the relationship between *HealthStatus* and lifestyle variables (*Smoking, Alcohol* and *Workout*) we also include interaction terms between the lifestyle variables and *HealthLiteracy* in later specifications.

In the case where our dependent variable is *HealthLiteracy*, which is a discrete variable, we estimate the regression via an ordered logit model and display the odds ratios. In the case where our dependent variable is *HealthStatus* we opt for an Ordinary Least Squares (OLS) estimation.

## Results

### Summary statistics and correlations

The following Table 1 presents the summary statistics of the variables that will be used in the regression analysis.

**Table 1:** Summary statistics of the variables of interest

**Variable**	**Obs**	**Mean**	**Std. Dev.**	**Min**	**Max**
*HealthLiteracy*	1526	2.359109	1.299485	0	4
*HealthStatus*	1526	77.2097	15.0409	15	100
*Male*	1526	0.45675	0.498289	0	1
*Income[<1,100]*	1526	0.29882	0.457891	0	1
*Income[1,100-2,200]*	1526	0.359764	0.480089	0	1
*Income[>2,200]*	1526	0.341416	0.47434	0	1
*Smoke*	1526	0.376147	0.484576	0	1
*Workout*	1526	0.634993	0.48159	0	1
*Alcohol*	1526	0.216252	0.411823	0	1

Health literacy study among university students, Greece, 2013.

The average respondent scored a *HealthLiteracy* grade of 2.4 indicating a fair to high health literacy grade. The average *HealthStatus* in our sample is 77.21 (out of a 100). This is a reasonably high score, but not surprising considering that all our respondents are in the age of 15 to 24. In terms of demographic characteristics 46% of our respondents are males. About 30% of respondents have a monthly family income of less than 1,100 euros, 36% are between 1,100 and 2,200 euros, while the rest 34% has a monthly income of more than 2,200 euros. In terms of health habits, 38% of the respondents answered that smoke while 63% work out at least twice a week. Finally, 22% responded that they consume alcohol on a daily or almost daily basis.

Table 2 shows the correlations across variables along with their statistical significance.

**Table 2:** Correlations across variables of interest

	**HealthLiteracy**	**HealthStatus**	**Male**	**Income[<1,100]**	**Income[1,100-2,200]**	**Income[>2,200]**	**Smoke**	**Workout**	**Alcohol**
*HealthLiteracy*	1								
*HealthStatus*	0.059**	1							
*Male*	-0.064**	0.005	1						
*Income[<1,100]*	-0.016	-0.081***	-0.058**	1					
*Income[1,100-2,200]*	-0.058**	0.023	-0.030	-0.489***	1				
*Income[>2,200]*	0.074***	0.055**	0.086***	-0.470***	-0.540***	1			
*Smoking*	-0.046*	-0.174***	0.067***	-0.013	-0.027	0.040	1		
*Workout*	0.098***	0.225***	0.042	-0.040	-0.007	0.046*	-0.204***	1	
*Alcohol*	-0.046*	-0.100***	0.154***	0.036	-0.042*	0.008	0.240***	-0.098***	1

Health literacy study among university students, Greece, 2013.

*Note*: Three stars (***) indicate statistical significance at 1% level, two stars (**) at 5% level, and one star (*) at 10% level.

The correlation between the status of health and health literacy grade is small (i.e. 0.06), though it is statistically significant at the 5% level. Health literacy is negatively associated with the gender (male). In terms of income, we observe that as family income increases so does the health literacy grade. Health habits are also significantly correlated with the health literacy grade. Smoking and consumption of alcohol are negatively associated with the health literacy score even though the relationship is significant only at the 10% level. In contrast working out has a positive correlation with health literacy and is statistically significant at the 1% level.

A somewhat different picture emerges with the correlations of health status with the rest of the variables. For example, health status has no significant association with the sex of the respondent. In terms of income, we observe that respondents with the lowest income are associated with lower health status. While there is no significant correlation between middle income respondents and health status, respondents with the highest income are associated with higher health status; a result opposite than the case of the lowest income respondents. Furthermore, health status is associated negatively with smoking and alcohol consumption while positively with working out. All the aforementioned associations with the health habits are significant at the 1% level.

Finally, it is interesting to examine the association across the demographic and health habit variables. This is a crucial stage in the analysis as it reveals whether these variables, which will be employed as regressors in the regression analysis, suffer from multicollinearity. The income variables naturally have high degree of association as they are mutually exclusive. However, note that only two of the three variables (i.e. *Income[<1,100], Income[1,100-2,200]*) will be employed in the regression analysis. Excluding these correlations, the highest correlation between these variables is 0.24 (alcohol and smoking). Therefore, we do not have any significant correlation between any of our variables, indicating lack of significant multicollinearity.

### Results of estimations

Table 3 examines how each variable is associated with the dependent variable health literacy grade (*HealthLiteracy*) by displaying results from an ordinary logistic regression. We display the odds ratios. *HealthStatus* does not seem to be associated significantly with *HealthLiteracy*. Males appear to score lower in *HealthLiteracy*. For instance, being a *Male* decreases his ordered log-odds of being in a higher *HealthLiteracy* by 1–0.786 = 21.4%.

**Table 3:** Factors associated with health literacy

	**Proportional odds ratio Health literacy**	**95% confidence intervals**
*HealthStatus*	1.003	(0.998 ; 1.009)
*Male*	0.786***	(0.654 ; 0.944)
*Income[<1,100]*	0.820*	(0.657 ; 1.023)
*Income[1,100 – 2,200]*	0.737***	(0.596 ; 0.912)
*Smoking*	0.936	(0.772 ; 1.134)
*Workout*	1.316***	(1.079 ; 1.605)
*Alcohol*	0.917	(0.727 ; 1.157)
*Constant*	3.505***	(1.799 ; 5.291)
*Observations*	1,526	

Results from an ordinal logistic regression. Health literacy study among university students, Greece, 2013.

*Note*: The specification is estimated via an Ordered Logit. Three stars (***) indicate statistical significance at 1% level and one star (*) at 10% level.

Lower and middle income levels are on average associated with lower *HealthLiteracy*. Indicatively, a family income of less than 1,100 Euros decreases his/her ordered log-odds of being in a higher *HealthLiteracy* by 26.3% compared with a student whose family income is more than 2,200 Euros. Results are analogous when comparing *Income[1,100-2,200]*, and *Income[>2,200]*.

Of the health habit variables, only *Workout* appears to have a significant association with *HealthLiteracy*. Students that work out increase their ordered log-odds of being in a higher *HealthLiteracy* by 31.6% than students who do not work out.

Table 4, Column 1 shows how the demographic characteristics, health habits and health literacy are associated with the perceived health status, which is now the dependent variable. Table 4 regressions have been estimated via OLS.

**Table 4:** Factors associated with health status

	**Endogenous variable: *healthStatus***	
	**(1)**	**(2)**
*HealthLiteracy*	0.361	1.199**
	(0.281)	(0.514)
*Male*	0.297	0.245
	(0.757)	(0.758)
*Income[<1,100]*	-2.676***	-2.784***
	(0.952)	(0.951)
*Income[1,100 – 2,200]*	-0.591	-0.610
	(0.887)	(0.888)
*Smoking*	-3.877***	-1.028
	(0.818)	(1.657)
*Workout*	5.849***	7.084***
	(0.818)	(1.611)
*Alcohol*	-1.830*	-0.922
	(1.040)	(2.066)
*SmokingxHealthLiteracy*		-1.188*
		(0.608)
*Workoutx HealthLiteracy*		-0.489
		(0.587)
*Alcoholx HealthLiteracy*		-0.402
		(0.769)
*Constant*	75.38***	73.39***
	(1.216)	(1.531)
		
*Observations*	1,526	1,526
*R-squared*	0.077	0.080

Results from Ordinary Least Squares Estimation. Health literacy study among university students, Greece, 2013.

*Note*: Both columns have been estimated via Ordinary Least Squares. Numbers in parentheses are robust standard errors. Three stars (***) indicate statistical significance at 1% level, two stars (**) at 5% level, and one star (*) at 10% level.

Students in the lowest income category have 2.7 less units of the perceived health status compared to the highest income category. The difference between the middle income and highest income category is neither large nor statistically significant. With respect to the health habits, students that smoke have health status lower by 3.9 units compared with students that do not smoke. Working out is associated with 5.85 higher health status. The coefficients of the two aforementioned variables are statistically significant at the 1% level. As expected, consumption of alcohol relates to lower health status by 1.83 units; however, the coefficient is less significant than the previous two behaviour variables.

The coefficient of *HealthLiteracy* is not statistically significant. In column 2 we include interaction terms of *HealthLiteracy* with the health habit variables. This estimation shows that *HealthLiteracy* of those that do not work out, do not consume alcohol nor smoking is associated positively with *HealthStatus*. Lastly, *HealthLiteracy* of those that smoke is associated negatively with *HealthStatus*. Finally there is no statistical difference of an additional contribution in the association of *HealthLiteracy* and *HealthStatus* of students that work out or consume alcohol.

## Discussion

Using a random sample of 1,526 university students in Greece, aged 18–24 years old, we first calculated the level of health literacy and its association with perceived health status, various demographics and health behaviours.

Summing up, we found that the health literacy level of the university students in our sample is of medium to high level and that their health status is very good. Additionally, economic factors, such as family income, demographic factors, such as gender, and health behaviours and risks, namely consumption of alcohol, smoking and physical workout are associated with the level of health literacy and health status of the participant.

While the results of the study are consistent with previous work in this area, several findings worth further comments.

The level of health literacy of the participant is positively and statistically associated with the level of income. Specifically, individuals with higher family income are more likely to score higher in the health literacy questions. The findings are consistent with those of HLS-EU Survey Report for Greece [29], but also with other studies [30-33]. Furthermore, gender matters. Being a male student is associated with lower health literacy. This might be surprising, as it contradicts with the general findings of other studies [20,29,34-39], but women in Greece tend to care more about dieting or due to parenting (child birth and care), therefore, appear to be also more literate in health matters. Among an individual’s health behaviours, only working out is associated with higher health literacy, while many studies have contradictory findings, noting that also other health behaviours and risks are associated with the health literacy level [4,12,14,20].

As of the health status of the participants, individuals with the lowest family income have lower health status than individuals with the highest family income. Almost all studies in the field have the same findings. Health behaviours have the anticipated significant relationships with health status: working out increases health status while smoking and alcohol decrease health status, common findings in almost all relevant studies. Family income and workout are the common factors that strongly relate with both health literacy and status of health of an individual. Recent studies have also include many other factors in the basket of those strongly associated with both health literacy level and health status [4,6,8,12,14,20,40-45].

Finally, we do not find a significant association between health status and health literacy, conflicting to the results of other studies [4,29,12,20] but making sense as our study is focusing on young people with *a priori* good health, as some other studies have also noted [19,21,29-31]. Nonetheless, the association of health literacy and health status varies by health habit groups; for instance health literacy of smokers is associated differently with health than health literacy of non-smokers.

Several limitations of the study are worth noting. First, though the students’ sample is of remarkable size and in was carried out in many universities and spread out in many geographical areas of the country, does not necessarily reflects the characteristics of the university students in Greece as a whole. Second, the research method for assessing the health literacy level focused on solely its functional aspect, using a short but convenient and comprehensive questionnaire. Nevertheless, a broader consensus for the best method to be adopted still lacking [3,4,7,15,23].

## Conclusions

In the European Union, one of the central objectives is to increase the ability of citizens to take better decisions about their health, by ensuring easy access to clear and reliable information on how to be in good health and about diseases and treatment options [29].

In this context, the Ministry of Education and Religion Affairs (MoE&RA) as well as the Ministry of Health (MoH) have set the promotion of the health literacy (especially in students), among their primary goals. So, the promotion of health literacy is mentioned repeatedly as an objective in the National Action Plan for Health Education Programs and the National Action Plan for Public Health 2008–2012 respectively [46,47]. Though, health promotion interventions in Greece include health literacy as one of the basic pillars of the Public Health policy, it is clear, that health literacy needs to become a key policy issue in Greece, mainly focusing in adolescents. This because in these young ages, healthy (or unhealthy) behaviours are established affecting the health through the life span.

## Competing interest

The authors declare that they have no competing interests.

## Authors’ contributions

AV and KD conceived the idea for the study, designed the protocol and supervised the performance of the study; KM performed the retrieval of the sample; AV and KD performed the preliminary and the final analyses; All authors discussed the interpretation of the results; AV and KD drafted the final report; all authors revised the article critically. All authors read and approved the final manuscript.
